# ATM Deficiency Results in Accumulation of DNA-Topoisomerase I Covalent Intermediates in Neural Cells

**DOI:** 10.1371/journal.pone.0058239

**Published:** 2013-04-23

**Authors:** Meryem Alagoz, Shih-Chieh Chiang, Abhishek Sharma, Sherif F. El-Khamisy

**Affiliations:** 1 Genome Damage and Stability Centre, School of Life Sciences, University of Sussex, Brighton, United Kingdom; 2 Department of Biochemistry, Faculty of Pharmacy, Ain Shams University, Cairo, Egypt; 3 Genome Centre, Helmy Institute for Medical Sciences, Zewail City, Giza, Egypt; University of Louisville, United States of America

## Abstract

Accumulation of peptide-linked DNA breaks contributes to neurodegeration in humans. This is typified by defects in tyrosyl DNA phosphodiesterase 1 (TDP1) and human hereditary ataxia. TDP1 primarily operates at single-strand breaks (SSBs) created by oxidative stress or by collision of transcription machinery with topoisomerase I intermediates (Top1-CCs). Cellular and cell-free studies have shown that Top1 at stalled Top1-CCs is first degraded to a small peptide resulting in Top1-SSBs, which are the primary substrates for TDP1. Here we established an assay to directly compare Top1-SSBs and Top1-CCs. We subsequently employed this assay to reveal an increased steady state level of Top1-CCs in neural cells lacking Atm; the protein mutated in ataxia telangiectasia. Our data suggest that the accumulation of endogenous Top1-CCs in *Atm*-/- neural cells is primarily due to elevated levels of reactive oxygen species. Biochemical purification of Top1-CCs from neural cell extract and the use of Top1 poisons further confirmed a role for Atm during the formation/resolution of Top1-CCs. Finally, we report that global transcription is reduced in *Atm*-/- neural cells and fails to recover to normal levels following Top1-mediated DNA damage. Together, these data identify a distinct role for ATM during the formation/resolution of neural Top1-CCs and suggest that their accumulation contributes to the neuropathology of ataxia telangiectasia.

## Introduction

Chromosomal single-strand breaks (SSBs) are one of the most abundant forms of endogenous DNA breakage. They can arise from various sources including the abortive activity of endogenous enzymes such as DNA topoisomerases. During its normal catalytic cycle, DNA Topoisomerase I (Top1) forms a transient and “reversible” intermediate where it becomes covalently attached to the 3′-terminus of a DNA nick, called Top1-cleavage complex (Top1-CC). If Top1-CCs encounter a DNA or RNA polymerase they become “irreversible”, resulting in Top1-SSBs or Top1-double strand breaks (DSBs) [1]. The rapid resealing of Top1-CCs can also be retarded by endogenous DNA base modifications, such as oxidation, alkylation, or base mismatch [2]. Cells have evolved specialized and partially overlapping mechanisms to repair this type of DNA breakage, including the nucleolytic cleavage of DNA or the hydrolytic cleavage of the phosphodiester bond between stalled Top1 and DNA. Defects in the latter activity have been associated with human hereditary ataxia and cerebellar degeneration, as illustrated by spinocerebellar ataxia with axonal neuropathy 1 (SCAN1) – mutated in tyrosyl DNA phosphodiesterase 1 (TDP1) [3]. The most striking neurological presentation in SCAN1 appears similar, if not identical, to that caused by deficiency of the protein mutated in A–T (Ataxia Telangiectasia mutated, ATM) where the cerebellum is a common target [4], [5], raising the intriguing possibility that the observed neural demise is caused by similar lesions and/or attenuation of a common pathway [6].

ATM regulates the function of an extensive network of downstream factors, in response to DNA damage. For example, ATM phosphorylates TDP1 and DNA polynucleotide kinase (PNK), which facilitates double-strand break repair and cellular survival following exposure to Top1 poisons [7]–[9]. In addition to its phosphorylation, TDP1 is a substrate for SUMOylation, which promotes its accumulation at sites of DNA damage and increases the rate of chromosomal SSB repair during transcription [10]. Top1 poisoning of non-cycling cells has also been shown to activate ATM [11], [12], however the consequences of ATM deficiency and the nature of DNA breaks that accumulate remain elusive. Here we developed a comet assay method that allows the direct comparison of Top1-CCs and Top1-SSBs, and consequently adopted this method to examine the nature of DNA damage that accumulates endogenously in the absence of ATM.

## Results and Discussion

**Figure 1:**
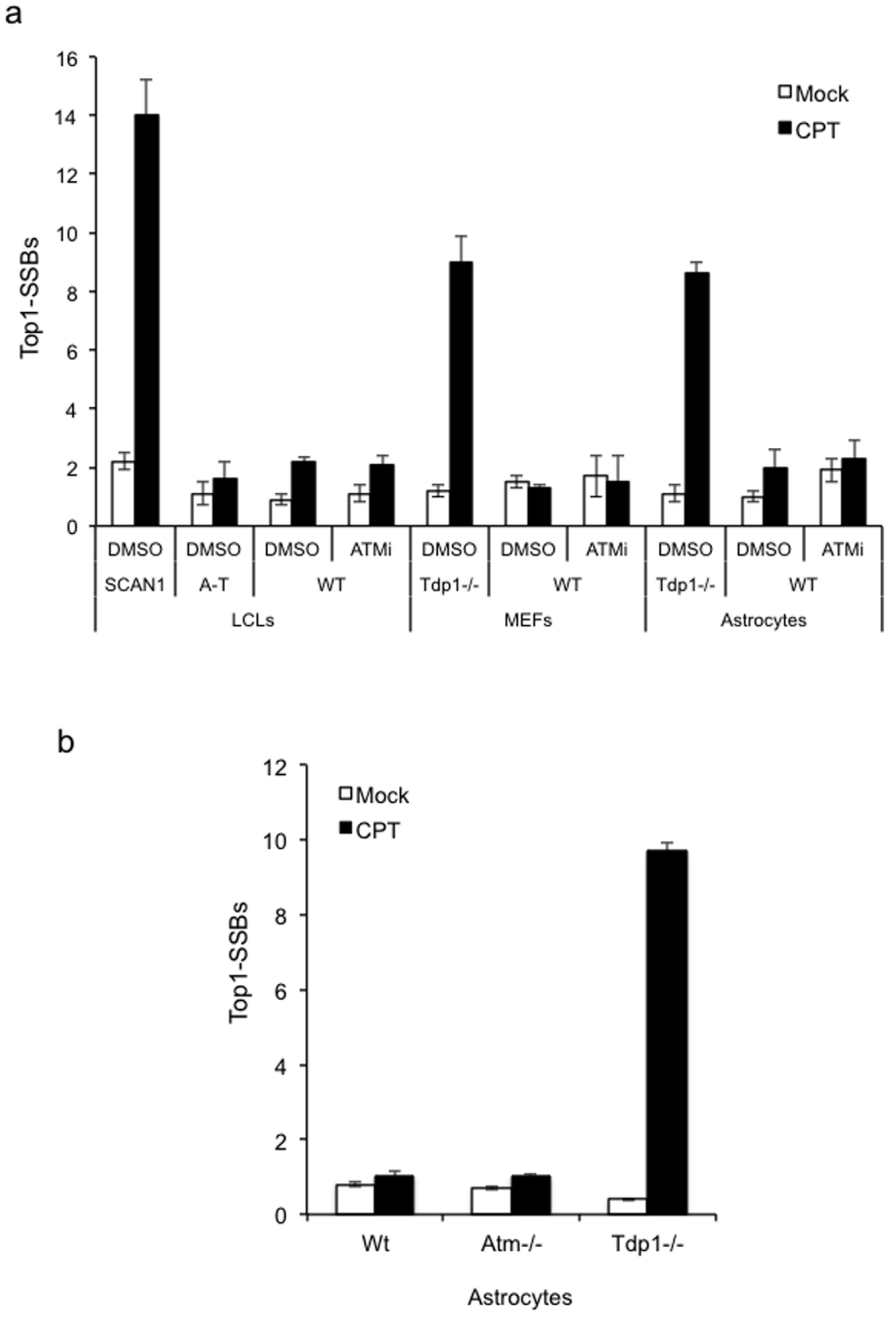
ATM deficiency does not impact on the accumulation of Top1-single-strand breaks. (**a**) Human lymphoblastoid cells (LCLs) derived from a normal individual ‘WT’, spinocerebellar ataxia with axonal neuropathy ‘SCAN1’, or ataxia telangiectasia ‘A–T’ patients, and mouse embryonic fibroblasts (MEFs) or quiescent cortical astrocytes from control ‘WT’ or *Tdp1-/-* mice were incubated with DMSO (Mock) or 30 µM camptothecin (CPT) for 40 min with or without pre-incubation with 10 µM ATM inhibitor KU-55933 (ATMi) for 2 hours at 37°C. Top1-single-strand breaks ‘Top1-SSBs’ were quantified by alkaline comet assays (ACAs). Mean tail moments were calculated for 50 cells/sample/experiment and data are the average of *n* = 3 biological replicates ± s.e.m. (**b**) Top1-SSBs were analysed in quiescent cortical astrocytes derived from wild-type ‘WT’, *Atm-/-* or *Tdp1-/-* mice following incubation with DMSO (Mock) or 30 µM camptothecin (CPT) and quantified as described above.

To examine the role of ATM during Topoisomerase I-mediated DNA single-strand break repair (SSBR) we incubated human lymphoblastoid cells (LCLs), murine embryonic fibroblasts (MEFs), or murine cortical astrocytes with the Top1 poison camptothecin (CPT) in presence or absence of the ATM inhibitor (ATMi) KU-55933 and measured DNA strand breaks by alkaline comet assays (Fig.1a). As predicted, cells deficient in TDP1 accumulated remarkably higher levels of Top1-SSBs during 40 min incubation with CPT whereas co-addition of ATMi did not affect the level of CPT-induced SSBs. This was not due to residual ATM activity since loss of ATM did not result in higher levels of Top1-SSBs in human cells or primary murine neural cells (Fig.1a&b).

In non-cycling cells oxidative stress and transcription are the primary sources for Top1-breaks and emerging evidence implicates PI3 kinases in transcriptional responses following DNA damage [13]–[15]. Notably, transcriptional arrest following Top1 poisons has also been shown to trigger the degradation of stalled Top1 from Top1-DNA cleavage complexes ‘Top1-CCs’ [16], [17], raising the possibility that proteasomal degradation of Top1 may be regulated by ATM to maintain transcriptional integrity. Crystal structure and cellular studies have shown that full-length Top1 at stalled Top1-CCs is first degraded by the proteasome to a small peptide, which then becomes accessible to TDP1 [18]–[20]. The proteasomal degradation process, however, cannot be examined by alkaline comet assays (ACA) since un-degraded ‘reversible’ Top1-CCs would not be visible. This is because CPT is washed immediately after treatment, thus any ‘reversible’ un-degraded Top1-CCs will re-ligate. In addition, DNA covalently bound to un-degraded Top1 would not be expected to produce a measurable tail upon electrophoresis. Indeed, this is illustrated by the absence of a measurable increase of breaks above background level in wild-type cells treated with CPT using the ‘classical’ ACAs (Fig. 1b).

**Figure 2:**
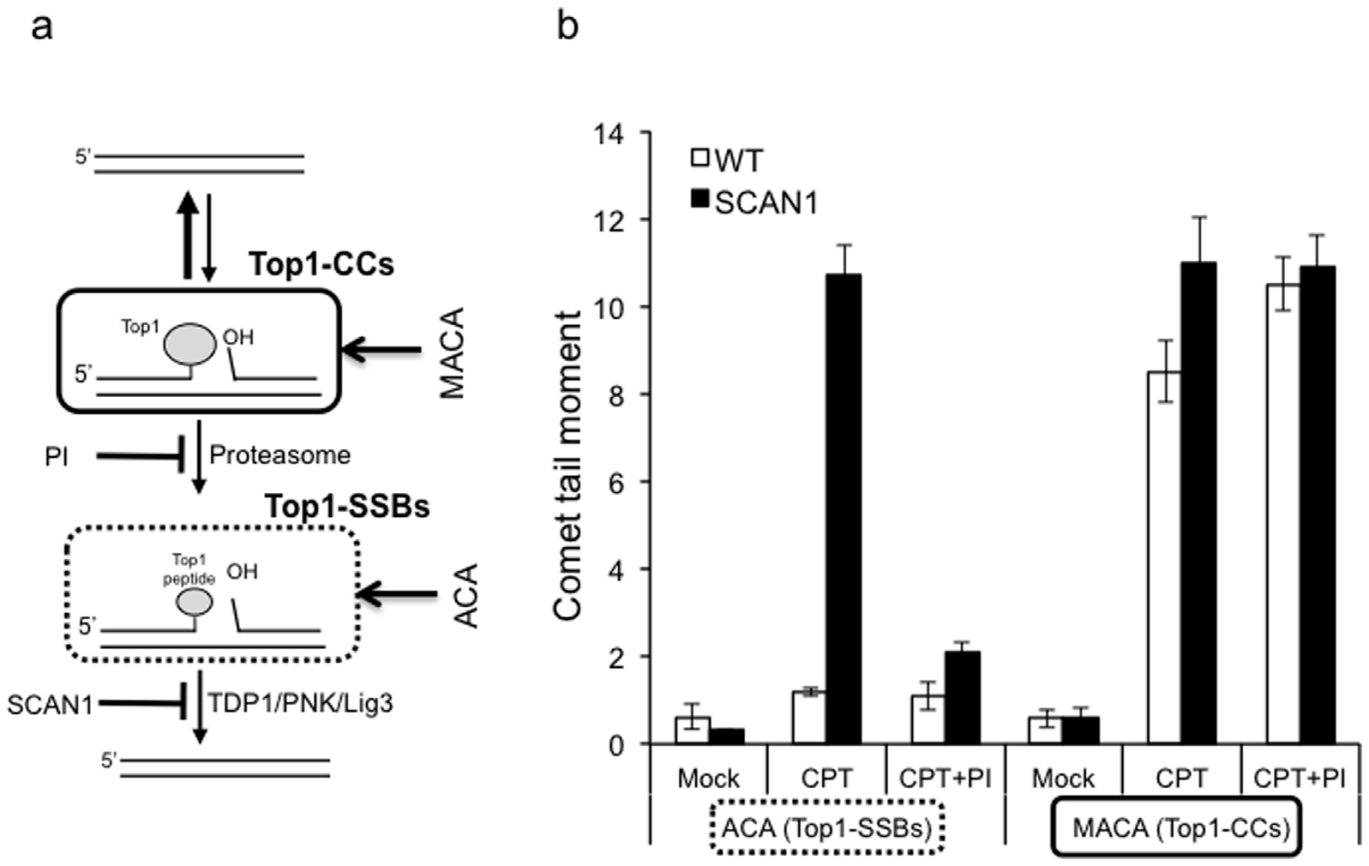
Modification of the alkaline comet assay uncovers un-degraded Top1-DNA cleavage complexes (Top1-CCs). (**a**) Scheme depicting the major differences between Top1-CCs and Top1-SSBs: Top1 relaxes DNA supercoiling by introducing a reversible nick to which Top1 becomes covalently attached (Top1-CCs). Stalling of Top1-CCs through collision with the transcription machinery or oxidative DNA damage triggers proteasomal degradation of Top1, resulting in Top1 single-strand breaks (Top1-SSBs). Repair of Top1-SSBs is initiated by removal of Top1 peptide by TDP1 followed by subsequent ligation. ***Note that un-degraded ‘Top1-CCs’ are not detected by the ‘classical’ alkaline comet assays (ACA) due to the reversible nature of these intermediates and the reduced ability of covalently bound Top1 on DNA to produce measurable tail upon electrophoresis.*** (**b**) Control ‘WT’ or SCAN1 LCLs ‘SCAN1’ harbouring the TDP1 catalytic mutation H493R were incubated with 20 µM camptothecin “CPT” with or without a prior 2-hr incubation with 30 µM proteasome inhibitor MG132 ‘PI’. Cells were divided into two fractions for the comparative detection of Top1-SSBs and Top1-CCs using the ACAs and modified ACAs ‘MACA’, respectively. Mean tail moments were calculated for 50 cells/sample/experiment and data are the average of *n* = 3 biological replicates ± s.e.m. ***Note that inhibiting the proteasome resulted in a reduction of Top1-SSBs (as measured by ACA) to near background levels with minimal impact on Top1-CCs (as measured by MACA).***

To uncover Top1-CCs and compare them directly with Top1-SSBs, we developed a modification of the ACA by conducting all steps prior to lysis at ambient temperature and keeping CPT throughout the analyses. In addition, we complemented the lysis step with proteinase K treatment to uncover Top1-CCs during electrophoresis (Fig. 2a). For optimisation experiments, we exploited SCAN1 lymphoblastoid cells (LCLs), which are easier to grow and manipulate than primary neural cultures. These cells harbour the TDP1 catalytic mutation H493R, which increases the formation of protein-linked DNA breaks. Treatment of SCAN1 cells with CPT resulted in ∼10-fold increase of SSBs compared to control cells (Fig. 2b, left). The repair of CPT-induced SSBs in TDP1 proficient cells is normally fast and thus low levels of SSBs were detected in control cells. Consistent with the requirement of proteasomal degradation of Top1 to uncover SSBs, inhibiting the proteasome with MG132 reduced SSBs to nearly background levels (Fig. 2b, left). In striking contrast to the ACA, the modified ACA (MACA) revealed ∼8-fold increase of Top1-CCs in wild-type cells (Fig. 2b, right). The nature of these structures was further confirmed by their persistence in presence of MG132, indicating that they are ‘un-degraded’ Top1-CCs.

**Figure 3:**
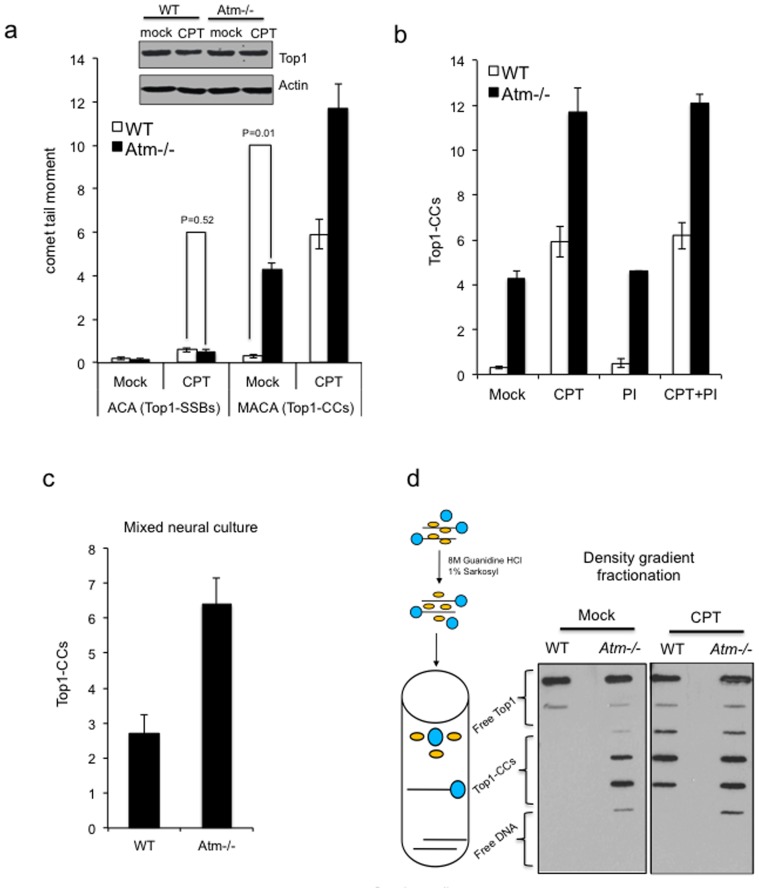
Loss of *Atm* results in accumulation of Top1-CCs in cortical neural cells. (**a**) Endogenous steady-state level of Top1-SSBs and Top1-CCs were quantified in quiescent wild type ‘WT’ and *Atm-/-* cortical astrocytes by ACAs and MACAs, respectively. Neural cells were subsequently subjected to 30 µM CPT for 40 min at 37 °C and the level of Top1-SSBs and Top1-CCs were quantified as above from 50 cells/sample/experiment. Data are the average of *n* = 3 biological replicates ± s.e.m. ***Inset***: astrocytes were incubated with DMSO ‘Mock’ or CPT ‘CPT’ and the expression of Top1 was measured by anti-Top1 immunoblotting. Anti-actin was employed as a loading control. (**b**) WT or *Atm-/-* quiescent astrocytes were mock incubated with DMSO ‘Mock’ or with the 30 µM CPT for 40 min at 37°C with or without a prior 2-hour incubation with the proteasome inhibitor MG132 ‘PI’. Top1-CCs were quantified by MACA from 50 cells/sample/experiment and the average of *n* = 3 biological replicates ± s.e.m is presented (**c**) Cortices from WT or Atm-/- mice were harvested at P6 and cells were dissociated and immediately subjected to MACA analyses. Data are the average of n = 3 biological replicates ± s.e.m. (**d**) **Left**: scheme depicting the biochemical fractionation of Top1-CCs. Blue circles are Top1 covalently bound to DNA (Top1-CCs) and yellow circles are Top1 non-covalently bound to DNA. **Right**: Wild-type ‘WT’ or *Atm-/-* quiescent cortical astrocytes were mock incubated or incubated with 30 µM CPT for 60 min at 37 °C. Neural cells were lysed in denaturing buffer and lysates fractionated on CsCl gradients. Fractions were slot blotted onto nitrocellulose and immunblotted with anti-Top1 monoclonal antibodies. A representative experiment from 3 biological replicates is shown. P values indicate the statistical difference between WT and *Atm-/-* cells (student t-test).

Having reached conditions that allow the direct comparison of Top1-SSBs and Top1-CCs, we next examined the role of ATM in controlling the steady-state level of the latter, in quiescent neural cells. In contrast to ACA, MACA revealed a significant increase (P = 0.006, t-test) of Top1-CCs in *Atm-/-* neural cells, even without exposure to exogenous sources of DNA damage (Fig. 3a). These observations are striking and suggest that Top1-DNA adducts arise endogenously in cells and that ATM deficiency results in their accumulation. Consistent with the latter, subsequent incubation with CPT led to a further increase of Top1-CCs in *Atm-/-* neural cells above that observed in control cells (Fig. 3a). Importantly, the difference in Top1-CCs was not due to differences in expression level of Top1 as shown by immunoblotting (Fig. 3a, inset) and was retained in the presence of proteasome inhibitors (Fig. 3b), confirming that they are ‘un-degraded’ Top1-CCs. In addition, experiments conducted directly on dissociated murine cortical neural cells also revealed higher levels of Top1-CCs in *Atm-/-* than control cells (Fig. 3c). Furthermore, the elevated level of Top1-CCs in *Atm-/-* neural cells was confirmed by an independent assay in which Top1-CCs were purified by density gradient fractionation and examined by anti-Top1 immunoblotting (Fig. 3d).

**Figure 4:**
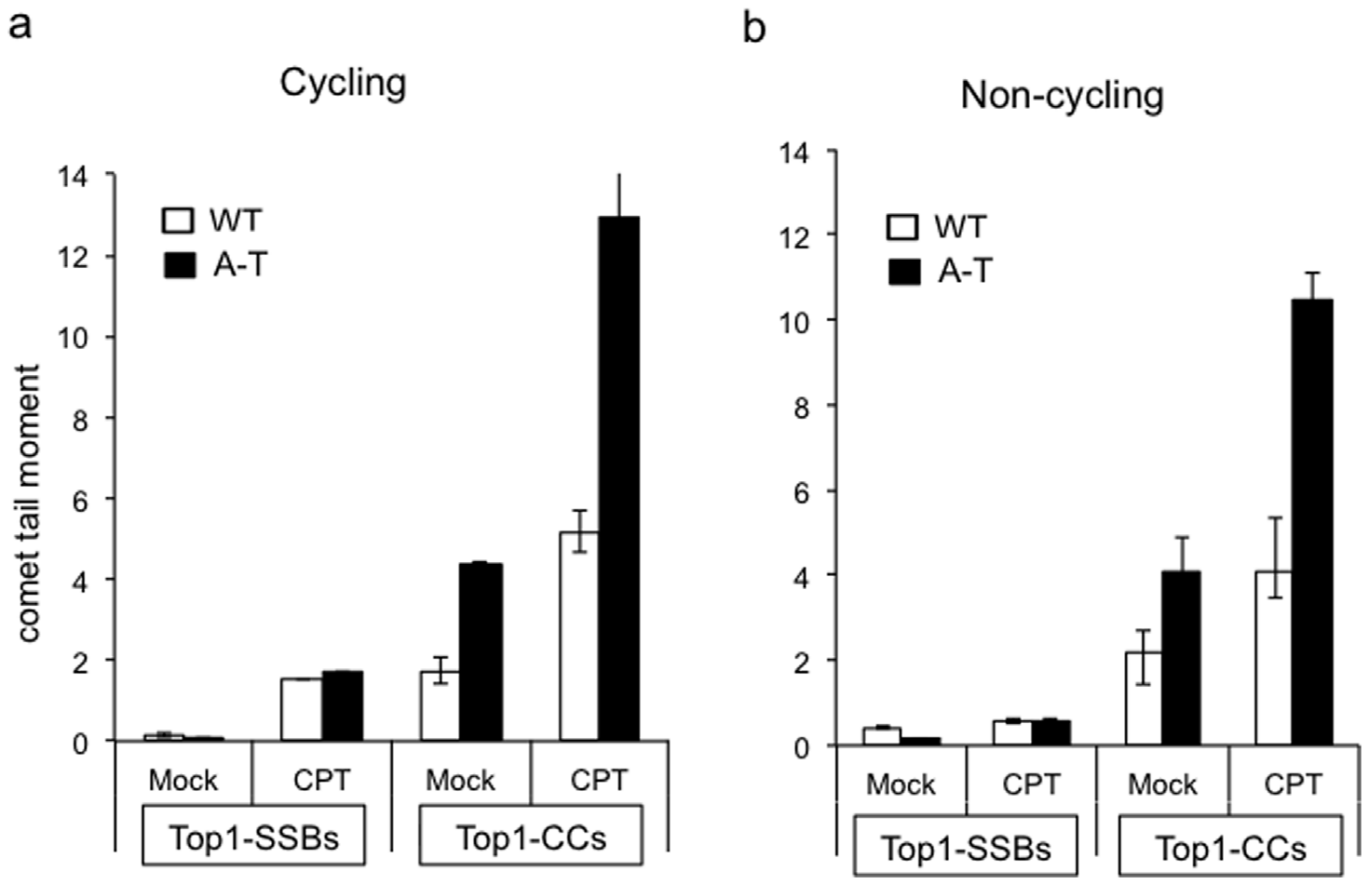
ATM deficiency results in accumulation of Top1-CCs in human cells. (**a**) Top1-SSBs and Top1-CCs were quantified in wild-type 1BR3 ‘WT’ or ATM deficient AT-1BR ‘A–T’ human primary fibroblasts by ACAs and MACAs, respectively. Cells were mock incubated with DMSO ‘Mock’ or with the 30 µM CPT for 40 min at 37°C and DNA strand breaks quantified from 50 cells/sample/experiment. Data are the average of *n* = 3 biological replicates ± s.e.m. (**b**) Primary human fibroblasts were grown to confluency and serum starved for 3 days, and Top1-SSBs/Top1-CCs were quantified as described in (a).

**Figure 5:**
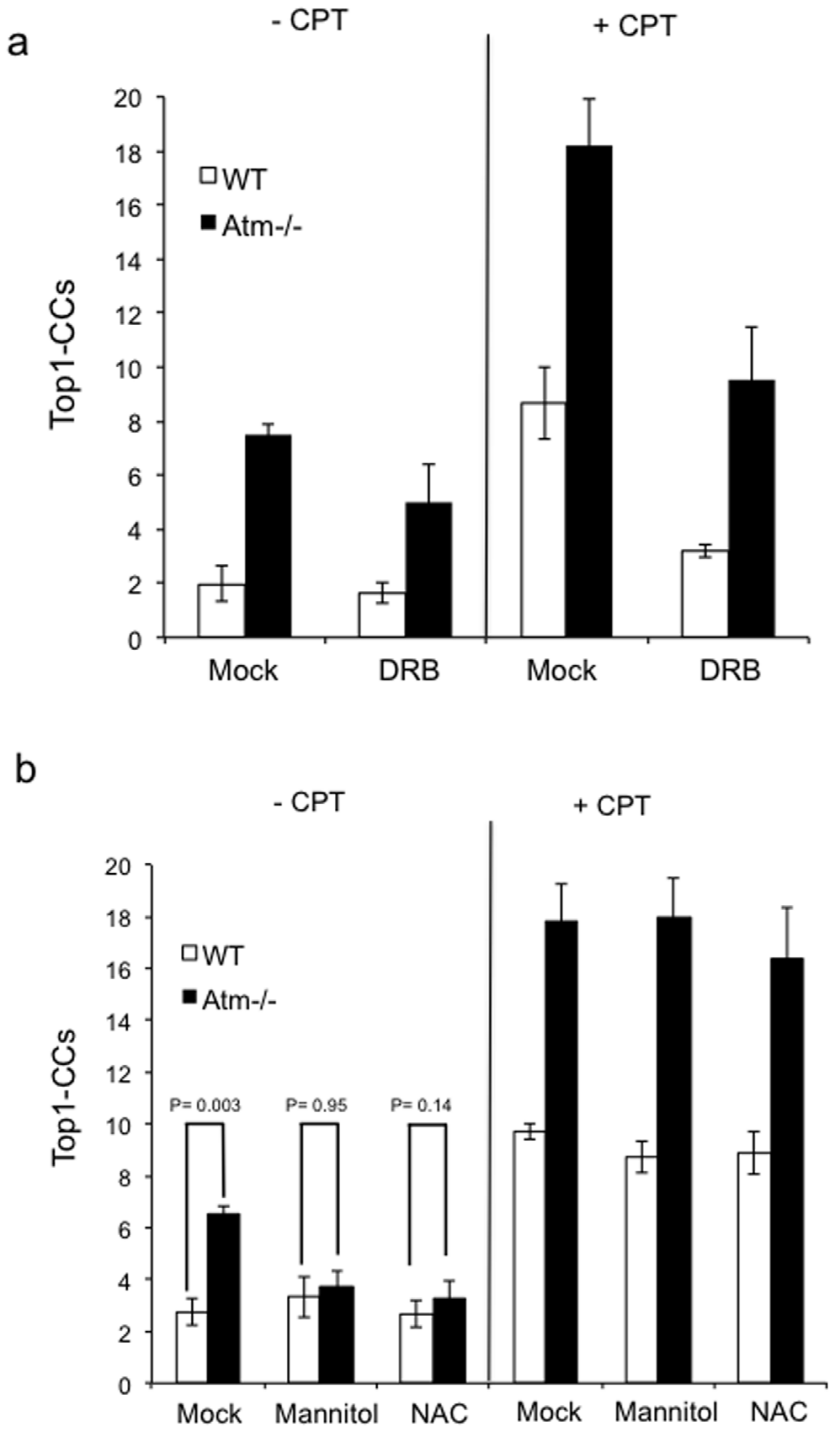
Reactive oxygen species scavengers reduce the accumulation of Top1-CCs in *Atm-/-* cells. (**a**) Top1-CCs were analysed in WT or *Atm-/-* quiescent astrocytes with and without incubation with 30 µM CPT ± a prior 2-hour incubation with 50 µM of the transcription inhibitor 5,6-dichloro-1-beta-D-ribofuranosylbenzimidazole ‘DRB’. Top1-CCs were quantified by MACA from 50 cells/sample/experiment and data represent the average of *n* = 3 biological replicates ± s.e.m. (**b**) Top1-CCs were analysed as in (a) with or without prior incubation with the reactive oxygen species (ROS) scavengers mannitol (50 mM) or N-Acetyl cysteine ‘NAC’ (10 mM) for 17-hours. 50 cells/sample/experiment were analysed and data represent the average of *n* = 3 biological replicates ± s.e.m. P values indicate the statistical difference between WT and *Atm-/-* cells (student t-test). ***Note that prior incubation with ROS scavengers reduces the endogenous level of Top1-CCs in Atm-/- cells to that observed in control cells*** **.**

How does Atm deficiency result in accumulation of Top1-CCs? We first repeated these experiments using human cells to examine whether this is due to a murine-specific role for Atm. Whilst Top1-SSBs accumulated to a similar extent in control and A–T human fibroblasts, the latter featured higher levels of Top1-CCs (Fig. 4a). This difference was largely unaffected by the replication status since serum starved non-cycling human primary A–T fibroblasts exhibited a similarly higher level of Top1-CCs (Fig. 4b). These data suggest that the role of ATM in regulating Top1-CCs is primarily controlled by replication-independent mechanisms. We next examined whether transcription plays a role in this process using quiescent primary neural cells. Pre-incubation with the transcription inhibitor 5,6-Dichloro-1-β-D-ribofuranosylbenzimidazole (DRB) resulted in a mild reduction of endogenous Top1-CCs in *Atm-/-* cells but their level was still higher than control cells (Fig. 5a; left), suggesting that other factors may account for this difference, at least at endogenous level of DNA damage. Consistent with a role for transcription during Top1-mediated DNA repair, DRB reduced the level of CPT-induced Top1-CCs in both control and *Atm-/- cells* (Fig. 5a, right).

We next considered the possibility that ATM deficiency may have caused the gradual accumulation of DNA damage in neural cells such as DNA nicks, abasic sites, or gaps – all of which have been shown to trap Top1 on DNA [2]. This would also be consistent with a role for ATM in regulating the level of reactive oxygen species (ROS) and anti-oxidant mechanisms [21]–[23]. However, if this is the case one would predict to observe higher levels of endogenous SSBs in *Atm-/-* neural cells by the ‘classical’ ACA, yet there was no significant difference observed (Fig.3a left; P = 0.52, t-test). It is unlikely that the ‘classical’ comet was not sensitive enough to measure subtle differences in SSBs since we have employed this technique to quantify small differences in SSB repair rates [24]. A more likely explanation is that the elevated level of ROS in *Atm-/-* cells may trap Top1 directly on DNA or may result in DNA breaks that are totally, or largely, ‘masked’ by Top1 and thus not detected by the ‘classical’ ACA. If this is true then pre-incubation with anti-oxidants should prevent the accumulation of Top1-CCs in *Atm-/-* neural cells. To test this possibility we compared Top1-CCs in neural cells pre-treated with two different ROS scavengers, mannitol [25] and N-acetyl cysteine (NAC) [26]. Intriguingly, pre-incubation with either mannitol or NAC prevented the accumulation of endogenous Top1-CCs in *Atm-/-* neural cells above control cells (Fig. 5b). In contrast, *Atm-/-* cells still retained higher levels of CPT-induced Top1-CCs in the presence of ROS scavengers.

Taken together, these data suggest that the elevated level of ROS in *Atm-/-* cells is a major source for the higher endogenous level of Top1-CCs and that ATM is likely to possess an additional, and perhaps more direct, role in resolving Top1-CCs particularly after challenging cells with CPT. The latter is supported by the observations that *Atm-/-* cells retained higher levels of CPT-induced Top1-CCs than control cells in presence of ROS scavengers. ATM may fulfil this role by promoting proteasomal degradation of Top1 at sites of ROS-induced Top1-CCs, in preparation for its subsequent removal by TDP1. There is precedence for the involvement of ATM during proteasomal processes. For example, ATM regulates subnuclear localization of TRF1 via a proteasome dependent mechanism [27]. Proteasome-mediated protein degradation has also been shown to be impaired in A–T cells and has been associated with suppression of the constitutively activated ISG15 pathway [28]. There is also evidence for persistent accumulation of non-degraded proteins in ATM deficient cells/tissues [29], [30].

ATM may impact on Top1 degradation directly by regulating its post-translational modification such as ubiquitylation and SUMOylation [17], [31], [32] or by controlling components of the ubiquitylation/SUMOylation machinery [17], [28]. For example, the E3-ubiquitin ligase Cullin 4B has recently been implicated in this process [33]. Alternatively ATM may promote the removal of full-length Top1 by facilitating the nucleolytic cleavage of DNA, releasing Top1 and a fragment of DNA (e.g. via the endonuclease activity of XPF [34]). By promoting the efficient resolution of Top1-mediated DNA damage, ATM maintains the integrity of transcriptome and viability of neural cells.

**Figure 6:**
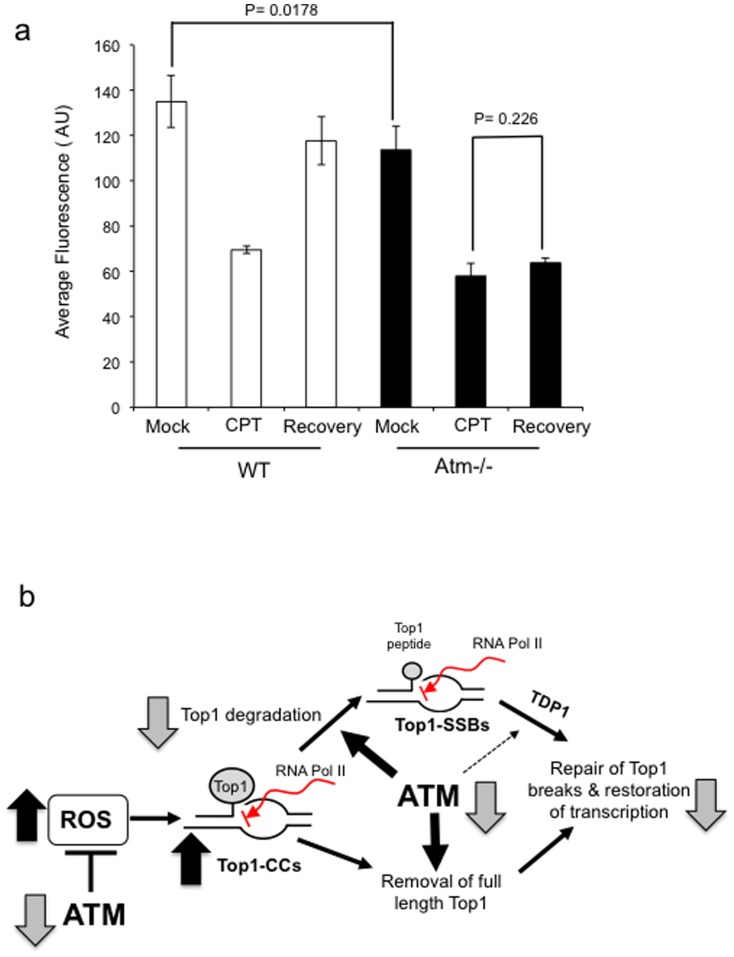
*Atm-/- neural cells* fail to recover transcription following Top1-mediated DNA damage. (a) Quiescent wild-type ‘WT’ or Atm-/- cortical astrocytes grown on coverslips were treated with 30 µM CPT for 1 hour and either harvested immediately after treatment or incubated in CPT-free media for a subsequent 3 hour to allow for transcription recovery. Cells were incubated with 0.1 mM 5-ethynl uridine (EU) for 30 min to label newly synthesized RNA, which was visualised by utilising the Click iT reaction with Alexa Flour azide 488. EU-labelled RNA was subjected to immuofluorescence analyses and data represent the average fluorescence signal (arbitrary units ‘AU’) from n = 3 biological replicates ± s.e.m, quantified from 200–300 cells using Corel Photo Draw software. P values indicate the statistical difference between the indicated bars (student t-test). (b) A model for the role of ATM during Top1-CC formation/resolution: ATM deficiency results in elevated levels of ROS, which leads to oxidative DNA breaks that are ‘masked’ from detection by the ‘classical’ comet due to trapping of Top1 on DNA, resulting in elevated steady state level of Top1-CCs. Stalling of Top1 on DNA can also occur through collision of Top1-CCs with elongating RNA polymerases ‘RNA Pol’. Top1 is first degraded by the proteasome to a small peptide, which is a substrate for the tyrosyl DNA phosphodiesterase activity of TDP1. We suggest that ATM facilitates the resolution of Top1-CCs by promoting Top1 degradation. ATM may also enhance the activity of one or more TDP1-independent processes to remove ‘un-degraded’ Top1 from DNA. Failure to remove Top1 from stalled Top1-CCs in ATM deficient cells results in a failure to maintain normal transcriptional activity following Top1-mediated DNA damage, and may contribute to the neuropathology of A–T.

Finally, if it is true that ATM maintains transcription integrity then ATM deficient neural cells should exhibit reduced global levels of transcription and reduced ability to maintain transcription following Top1-mediated DNA damage. To test this, we compared wild-type and *Atm-/-* neural cells for their ability to recover transcription following CPT treatment. Consistent with the increased level of endogenous Top1-CCs in *Atm-/-* cells, they exhibited subtle but consistent reduction of global transcription (Fig. 6a; P = 0.017). Whilst CPT treatment led to a marked reduction of transcription, subsequent incubation in CPT-free media restored transcription to nearly background levels in control but not in *Atm-/-* cells (Fig. 6a; P = 0.226). These data suggest that ATM is required for the maintenance of transcription following Top1-mediated DNA damage.

In summary, we identified a role for ATM during the formation/resolution of Top1-mediated DNA breaks, which is distinct from TDP1. ATM deficiency results in elevated levels of ROS, which consequently leads to higher Top1-CCs. This is further compounded by a more direct role for ATM during the resolution of Top1-CCs, which is likely mediated by proteasomal degradation of Top1 (Fig.6b). Accumulation of Top1-linked DNA breaks interferes with transcription and may contribute to the neuropathology of A-T, and perhaps other neurological disease.

## Materials and Methods

### Cell culture

Human 1BR3, AT-1BR fibroblasts [35], [36], or LCLs were cultured in Dulbecco's modified essential medium or RPMI (Gibco, Invitrogen), respectively. Media were supplemented with 10% fetal calf serum (FCS), 2 mM L-glutamine, 100 U/ml penicillin, and 100 µg/ml streptomycin. *Tdp1+/−* animals were mated and genotyping for the mutant *Tdp1* allele was performed on cell pellets, essentially as described [37]. *Atm+/−* mice were generated by the A. Wynshaw-Boris laboratory [38] and obtained from the Jackson Labs (stock ID: 002753). PCR primers were designed for genotyping *Atm-/-* mice. Primer pairs Atm-F1 (5′-AAACCGACTTCTGTCAGATGTTGC-3′) and Atm-R1 (5′-TTTGCAGGAGTTGCTGAGCG-3′) were used to identify the wild-type *Atm* allele. Atm-F2 (5′-GACTTCTGTCAGATGTTGCTGCC-3′) and Atm-R2 (5′-GGGTGGGATTAGATAAATGCCTG-3′) were used to identify the knockout allele in 35 cycles of 94°C for 20 sec, 58°C for 30 sec, and 72°C for 35 sec. The Atm-F1 and Atm-R1 pair generates a 152-bp PCR product, and the Atm-F2 and Atm-R2 pair generates a 441-bp PCR product. For the generation of primary cortical neural cells, mice were housed at the school of life sciences University of Sussex and maintained in accordance with the institutional animal care and ethical committee of the University of Sussex. Mice with the appropriate genotype were mated and murine astrocytes prepared from P3–P4 brains. Cortices were obtained from young pups by decapitation, dissociated by passage through a 5 ml pipette, and cells were either subjected directly to analyses by comet assays or re-suspended in Dulbecco's modified Eagle's medium and Ham's nutrient mixture F-12 (1∶1 DMEM/F12; Gibco-BRL) supplemented with 10% fetal calf serum, 1× glutamax, 100 U/ml penicillin, 100 µg/ml streptomycin, and 20 ng/ml epidermal growth factor (Sigma). Primary astrocytes were established in Primeria T-25 tissue culture flasks (Falcon) at 37°C in a humidified oxygen-regulated (5%) incubator. Once established, cells were maintained at 5% oxygen unless stated otherwise, grown to confluency and deprived from growth factors.

### Measurement of Top1-SSBs by ACAs

Top1 single-strand breaks (Top1-SSBs) were analysed by alkaline comet assays (ACA) [24]. Following incubation with DMSO or 30 µM CPT for 40 min, cells were washed 3× with PBS, suspended in pre-chilled PBS and mixed with equal volume of 1.2% low-gelling-temperature agarose (Sigma, type VII) maintained at 42°C. Cell suspension was immediately layered onto pre-chilled frosted glass slides (Fisher) pre-coated with 0.6% agarose and maintained in the dark at 4°C until set, and for all further steps. Slides were immersed in pre-chilled lysis buffer (2.5 M NaCl, 10 mM Tris-HCl, 100 mM EDTA pH 8.0, 1% Triton X-100, 1% DMSO; pH 10) for 1 hr, washed with pre-chilled distilled water (2×10 min), and placed for 45 min in pre-chilled alkaline electrophoresis buffer (50 mM NaOH, 1 mM EDTA, 1% DMSO). Electrophoresis was then conducted at 1V/cm for 25 min, followed by neutralization in 400 mM Tris-HCl pH 7.0 for 1 hr. Finally, DNA was stained with Sybr Green I (1∶10,000 in PBS) for 30 min. Average tail moments from 50 cells/sample were quantified from blindly coded slides using Comet Assay IV software (Perceptive Instruments, UK).

### Measurement of Top1-cleavage complexes (Top1-CCs) by MACAs

Top1-CCs were analysed by a modified version of the alkaline comet assay (MACA). Following incubation with DMSO or 30 µM CPT for 40 min, cells were washed 3× with PBS supplemented with DMSO or 50 µM CPT, at ambient temperature. Where indicated, cells were pre-incubated with 50 mM mannitol or 10 mM N-Acetyl cysteine (NAC) for 17 hrs, or with 50 µM of the transcription inhibitor 5,6-dichloro-1-beta-D-ribofuranosylbenzimidazole (DRB) for 2 hrs. Cells were finally suspended in PBS containing DMSO or 100 µM CPT supplemented with 800 µg/ml proteinase K (Sigma) and kept at ambient temperature. Cell suspension was mixed with equal volume of 1.2% low-gelling-temperature agarose (Sigma, type VII) maintained at 42°C, to bring the final CPT concentration to 50 µM. Cell suspension was immediately layered onto frosted glass slides (Fisher) pre-coated with 0.6% agarose and maintained in the dark at ambient temperature until set. Slides were immersed in lysis solution (400 µg/ml proteinase K, 50 µM CPT, 10 mM Tris-HCl, 100 mM EDTA pH 8.0, 1% Triton X-100, 1% DMSO; pH 10) for 1 hr at 40°C. Lysis solution was additionally supplemented with 2.5 M NaCl and left for a further 3 hrs at 40°C. Cells were immediately immersed in alkaline electrophoresis buffer (50 mM NaOH, 1 mM EDTA, 1% DMSO) and electrophoresis was conducted at 1 V/cm for 25 min, followed by neutralization in 400 mM Tris-HCl pH 7.0 for 1 hr. Finally, DNA was stained with Sybr Green I (1∶10,000 in PBS) for 30 min. Average tail moments from 50 cells/sample were quantified from blindly coded slides using Comet Assay IV software (Perceptive Instruments, UK).

### Measurement of Top1-CCs by biochemical fractionation and immunoblotting

Top1 protein–DNA complexes were detected as previously described [19]. Briefly, neural cells (2×10^6^) were mock treated or incubated in 30 µM CPT for 1 hr at 37 °C followed by lysis in 1% sarcosyl, 8 M guanidine HCl, 30 mM Tris pH 7.5 and 10 mM EDTA. Cell lysates were then incubated at 70°C for 15 min to remove all non-covalently bound proteins from DNA. Cell lysates were then loaded on a cesium chloride (CsCl) step gradient (5 ml total volume) and centrifuged at 75,600×g at 25°C for 24 hr to separate free proteins from DNA. Ten consecutive 0.5 ml fractions were collected and slot blotted onto Hybond-C membrane (Amersham). To ensure equal DNA loading, the DNA concentration in each extract was determined fluorimetrically using PicoGreen (Molecular Probes/Invitrogen). Covalent Top1–DNA complexes were then detected by immunoblotting with anti-Top1 monoclonal antibodies (Santa Cruz) and visualised by chemiluminescence.

### Measurement of global RNA transcription

Primary astrocytes were grown on coverslips for 48–72 hr in complete media deprived of growth factors. The Click-iT RNA Alaxa Flour 488 imaging Kit (Invitrogen) was employed according to manufacture's instructions to quantify global RNA transcription. Briefly, cells were treated with 30 µM CPT for 1 hr and either harvested immediately after treatment or washed twice with serum-free medium and incubated in CPT-free media for a subsequent 3 hr to allow for transcription recovery. Cells were incubated with 0.1 mM 5-ethynl uridine (EU) for 30 min in presence or absence of CPT to label newly synthesized RNA. Cells were washed with PBS, fixed with 3.7% formaldehyde for 15 min, and permeabilized with 0.5% Triton-X100 for 20 min at room temperature. Cells were then incubated with Click iT reaction cocktail containing Click-iT additive and Alexa Flour azide 488 for 30 min at 37°C. Following washing with PBS containing 3% BSA, cells were mounted on glass slides using a DAPI-containing antifade media. RNA labeled with EU was then subjected to immuofluorescence analyses. Average fluorescence signal was quantified from 200–300 cells using Corel Photo Draw software.

## References

[1] Pommier Y, Redon C, Rao VA, Seiler JA, Sordet O, et al (2003) Repair of and checkpoint response to topoisomerase I-mediated DNA damage. Mutat Res 532: 173–203. 14643436 10.1016/j.mrfmmm.2003.08.016

[2] Pourquier P, Pilon AA, Kohlhagen G, Mazumder A, Sharma A, et al (1997) Trapping of mammalian topoisomerase I and recombinations induced by damaged DNA containing nicks or gaps. Importance of DNA end phosphorylation and camptothecin effects. J Biol Chem 272: 26441–26447. 9334220 10.1074/jbc.272.42.26441

[3] Takashima H, Boerkoel CF, John J, Saifi GM, Salih MAM, et al (2002) Mutation of TDP1, encoding a topoisomerase I–dependent DNA damage repair enzyme, in spinocerebellar ataxia with axonal neuropathy. Nat Genet 32: 267–272. 12244316 10.1038/ng987

[4] Biton S, Dar I, Mittelman L, Pereg Y, Barzilai A, et al (2006) Nuclear ataxia-telangiectasia mutated (ATM) mediates the cellular response to DNA double strand breaks in human neuron-like cells. J Biol Chem 281: 17482–17491. 16627474 10.1074/jbc.M601895200

[5] El-Khamisy SF (2011) To live or to die: a matter of processing damaged DNA termini in neurons. EMBO Mol Med 3: 78–88. 21246735 10.1002/emmm.201000114 PMC3377058

[6] El-Khamisy SF (2003) A requirement for PARP-1 for the assembly or stability of XRCC1 nuclear foci at sites of oxidative DNA damage. Nucleic Acids Res 31: 5526–5533. 14500814 10.1093/nar/gkg761 PMC206461

[7] Chiang S-C, Carroll J, El-Khamisy SF (2010) TDP1 serine 81 promotes interaction with DNA ligase IIIalpha and facilitates cell survival following DNA damage. Cell Cycle 9: 588–595. 20009512 10.4161/cc.9.3.10598

[8] Das BB, Antony S, Gupta S, Dexheimer TS, Redon CE, et al (2009) Optimal function of the DNA repair enzyme TDP1 requires its phosphorylation by ATM and/or DNA-PK. EMBO J 28: 3667–3680. 19851285 10.1038/emboj.2009.302 PMC2790489

[9] Segal-Raz H, Mass G, Baranes-Bachar K, Lerenthal Y, Wang S-Y, et al (2011) ATM-mediated phosphorylation of polynucleotide kinase/phosphatase is required for effective DNA double-strand break repair. EMBO reports 12: 713–719. 21637298 10.1038/embor.2011.96 PMC3128972

[10] Hudson JJR, Chiang S-C, Wells OS, Rookyard C, El-Khamisy SF (2012) SUMO modification of the neuroprotective protein TDP1 facilitates chromosomal single-strand break repair. Nature Communications 3: 733–13. 10.1038/ncomms1739 PMC3316882 22415824

[11] Sordet O, Redon CE, Guirouilh-Barbat J, Smith S, Solier S, et al (2009) Ataxia telangiectasia mutated activation by transcription- and topoisomerase I-induced DNA double-strand breaks. EMBO reports 10: 887–893. 19557000 10.1038/embor.2009.97 PMC2726680

[12] Sakasai R, Teraoka H, Takagi M, Tibbetts RS (2010) Transcription-dependent activation of ataxia telangiectasia mutated prevents DNA-dependent protein kinase-mediated cell death in response to topoisomerase I poison. J Biol Chem 285: 15201–15208. 20304914 10.1074/jbc.M110.101808 PMC2865312

[13] Chamberlain SEL, González-González IM, Wilkinson KA, Konopacki FA, Kantamneni S, et al (2012) SUMOylation and phosphorylation of GluK2 regulate kainate receptor trafficking and synaptic plasticity. Nat Neurosci 15: 845–852. 22522402 10.1038/nn.3089 PMC3435142

[14] Baranello L, Bertozzi D, Fogli MV, Pommier Y, Capranico G (2010) DNA topoisomerase I inhibition by camptothecin induces escape of RNA polymerase II from promoter-proximal pause site, antisense transcription and histone acetylation at the human HIF-1alpha gene locus. Nucleic Acids Res 38: 159–171. 19854946 10.1093/nar/gkp817 PMC2800211

[15] Shanbhag NM, Rafalska-Metcalf IU, Balane-Bolivar C, Janicki SM, Greenberg RA (2010) ATM-dependent chromatin changes silence transcription in cis to DNA double-strand breaks. Cell 141: 970–981. 20550933 10.1016/j.cell.2010.04.038 PMC2920610

[16] Fu JC, Gruenwedel DW (1976) Salt effects on the denaturation of DNA. V. Preferential interactions of native and denatured calf thymus DNA in Na2SO4 solutions of varying ionic strength. Biopolymers 15: 265–282. 2350 10.1002/bip.1976.360150205

[17] Lin CP, Ban Y, Lyu YL, Desai SD, Liu LF (2008) A ubiquitin-proteasome pathway for the repair of topoisomerase I-DNA covalent complexes. J Biol Chem 283: 21074–21083. 18515798 10.1074/jbc.M803493200 PMC2475699

[18] Interthal H, Champoux JJ (2011) Effects of DNA and protein size on substrate cleavage by human tyrosyl-DNA phosphodiesterase 1. Biochem J 436: 559–566. 21463258 10.1042/BJ20101841 PMC3151729

[19] El-Khamisy SF, Hartsuiker E, Caldecott KW (2007) TDP1 facilitates repair of ionizing radiation-induced DNA single-strand breaks. DNA Repair (Amst) 6: 1485–1495. 17600775 10.1016/j.dnarep.2007.04.015

[20] Davies DR, Interthal H, Champoux JJ, Hol WGJ (2002) The crystal structure of human tyrosyl-DNA phosphodiesterase, Tdp1. Structure/Folding and Design 10: 237–248. 11839309 10.1016/s0969-2126(02)00707-4

[21] Ito K, Hirao A, Arai F, Matsuoka S, Takubo K, et al (2004) Regulation of oxidative stress by ATM is required for self-renewal of haematopoietic stem cells. Nature 431: 997–1002. 15496926 10.1038/nature02989

[22] Okuno Y, Nakamura-Ishizu A, Otsu K, Suda T, Kubota Y (2012) Pathological neoangiogenesis depends on oxidative stress regulation by ATM. Nat Med: doi: 10.1038/nm.2846. 10.1038/nm.2846 22797809

[23] Guo Z, Kozlov S, Lavin MF, Person MD, Paull TT (2010) ATM activation by oxidative stress. Science 330: 517–521. 20966255 10.1126/science.1192912

[24] Breslin C, Clements PM, El-Khamisy SF, Petermann E, Iles N, et al (2006) Measurement of chromosomal DNA single-strand breaks and replication fork progression rates. Meth Enzymol 409: 410–425. 16793415 10.1016/S0076-6879(05)09024-5

[25] Zhang C, Gong Y, Ma H, An C, Chen D, et al (2001) Reactive oxygen species involved in trichosanthin-induced apoptosis of human choriocarcinoma cells. Biochem J 355: 653–661. 11311127 10.1042/bj3550653 PMC1221780

[26] Kwak Y-D, Wang B, Li JJ, Wang R, Deng Q, et al (2012) Upregulation of the E3 ligase NEDD4-1 by oxidative stress degrades IGF-1 receptor protein in neurodegeneration. Journal of Neuroscience 32: 10971–10981. 22875931 10.1523/JNEUROSCI.1836-12.2012 PMC3681290

[27] McKerlie MA, Lin S, Zhu X-D (2012) ATM regulates proteasome-dependent subnuclear localization of TRF1, which is important for telomere maintenance. Nucleic Acids Res. 40(9): 3975–89. 10.1093/nar/gks035 PMC3351164 22266654

[28] Wood LM, Sankar S, Reed RE, Haas AL, Liu LF, et al (2011) A Novel Role for ATM in Regulating Proteasome-Mediated Protein Degradation through Suppression of the ISG15 Conjugation Pathway. PLoS ONE 6: e16422. 21298066 10.1371/journal.pone.0016422 PMC3027683

[29] Eilam R, Peter Y, Groner Y, Segal M (2003) Late degeneration of nigro-striatal neurons in ATM-/- mice. Neuroscience 121: 83–98. 12946702 10.1016/s0306-4522(03)00322-1

[30] Agamanolis DP, Greenstein JI (1979) Ataxia-telangiectasia. Report of a case with Lewy bodies and vascular abnormalities within cerebral tissue. J Neuropathol Exp Neurol 38: 475–489. 224149 10.1097/00005072-197909000-00003

[31] Mao Y, Sun M, Desai SD, Liu LF (2000) SUMO-1 conjugation to topoisomerase I: A possible repair response to topoisomerase-mediated DNA damage. Proc Natl Acad Sci USA 97: 4046–4051. 10759568 10.1073/pnas.080536597 PMC18143

[32] Yang M, Hsu CT, Ting CY, Liu LF, Hwang J (2006) Assembly of a polymeric chain of SUMO1 on human topoisomerase I in vitro. J Biol Chem 281: 8264–8274. 16428803 10.1074/jbc.M510364200

[33] Kerzendorfer C, Whibley A, Carpenter G, Outwin E, Chiang S-C, et al (2010) Mutations in Cullin 4B result in a human syndrome associated with increased camptothecin-induced topoisomerase I-dependent DNA breaks. Hum Mol Genet 19: 1324–1334. 20064923 10.1093/hmg/ddq008 PMC2838540

[34] Vance JR, Wilson TE (2002) Yeast Tdp1 and Rad1-Rad10 function as redundant pathways for repairing Top1 replicative damage. Proc Natl Acad Sci USA 99(21): 13669–13674. 12368472 10.1073/pnas.202242599 PMC129737

[35] Stiff T, Reis C, Alderton GK, Woodbine L, O'Driscoll M, et al (2005) Nbs1 is required for ATR-dependent phosphorylation events. EMBO J 24: 199–208. 15616588 10.1038/sj.emboj.7600504 PMC544916

[36] Taylor AM, Harnden DG, Arlett CF, Harcourt SA, Lehmann AR, et al (1975) Ataxia telangiectasia: a human mutation with abnormal radiation sensitivity. Nature 258: 427–429. 1196376 10.1038/258427a0

[37] El-Khamisy SF, Katyal S, Patel P, Ju L, McKinnon PJ, et al (2009) Synergistic decrease of DNA single-strand break repair rates in mouse neural cells lacking both Tdp1 and aprataxin. DNA Repair (Amst) 8: 760–766. 19303373 10.1016/j.dnarep.2009.02.002 PMC2693503

[38] Barlow C, Hirotsune S, Paylor R, Liyanage M, Eckhaus M, et al (1996) Atm-deficient mice: a paradigm of ataxia telangiectasia. Cell 86: 159–171. 8689683 10.1016/s0092-8674(00)80086-0

